# HL-TRP channel is required for various repellents for the parthenogenetic *Haemaphysalis longicornis*

**DOI:** 10.1186/s13071-025-06776-1

**Published:** 2025-04-14

**Authors:** Ceyan Kuang, Jie Cao, Yongzhi Zhou, Houshuang Zhang, Yanan Wang, Jinlin Zhou

**Affiliations:** Key Laboratory of Animal Parasitology of Ministry of Agriculture, Shanghai Veterinary Research Institute, Chinese Academy of Agricultural Sciences, Shanghai, 200241 China

**Keywords:** HL-TRP channel, Icaridin, DEET, Cinnamaldehyde, Olfaction

## Abstract

**Background:**

Ticks can transmit a wide range of pathogens that endanger human and animal health. Although repellents are commonly used for tick control, understanding their mechanisms aren't  complete.

**Methods:**

The repellent effects of N, N-diethyl-meta-toluamide (DEET); sec-butyl 2-(2-hydroxyethyl) piperidine-1-carboxylate (icaridin); N, N-diethyl-3-methylbenzamide (IR3535); and cinnamaldehyde on the parthenogenetic tick *Haemaphysalis longicornis* at the nymph stage were assessed using Y-tubes. The involvement of transient receptor potential (*HL-TRP*) channel molecules in the repellent mechanism was investigated through in situ hybridization, subcellular localization, real-time fluorescence quantitative polymerase chain reaction (PCR), RNA interference, and electroantennography. In addition, the binding affinity of HL-TRP molecules to repellents was predicted using AlphaFold3.

**Results:**

DEET, icaridin, IR3535, and cinnamaldehyde have been shown to effectively repel nymphs. HL-TRP channel is shared among various arthropods, particularly several species of ticks. It is localized to the cell membrane and Haller’s organ. Moreover, microinjection of double-stranded RNA elicited tick repellency behavior, and the electroantennogram responses to those repellents were significantly decreased. The TYR783 site was proposed as an essential binding site to establish hydrogen bonds with icaridin, DEET, and cinnamaldehyde.

**Conclusions:**

This exploration of ticks and repellents found that HL-TRP channel functions as a chemosensory receptor for repellents and, thereby, mediates avoidance behavior.

**Graphical Abstract:**

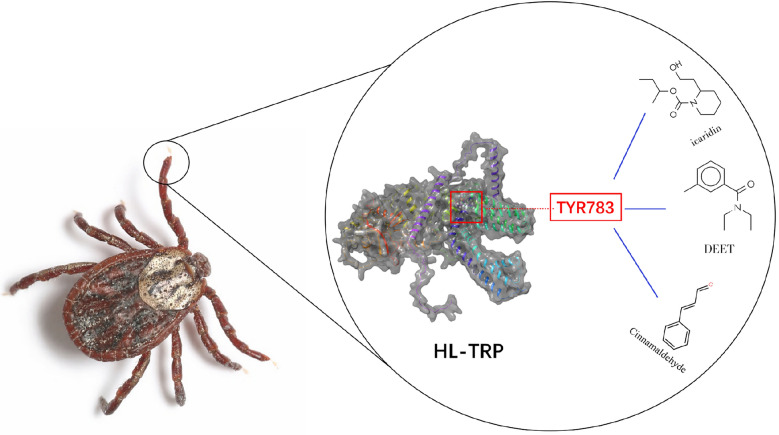

**Supplementary Information:**

The online version contains supplementary material available at 10.1186/s13071-025-06776-1.

## Background

Ticks are blood-sucking arthropod vectors that are capable of parasitizing a wide range of vertebrates. Ticks not only endanger the health of their hosts by feeding on blood but also pose a significant threat through the transmission of pathogens [1]. The life cycle of ticks comprises four stages: eggs, larvae, nymphs, and adults, with the duration of each stage ranging from a few months to several years [2, 3]. Larvae serve as important vectors of transovarial transmission of pathogens, while nymphs and adults frequently harbor a variety of pathogens that can infect their hosts. Compared with larvae and adults, nymphs are more susceptible to various pathogens and exhibit higher population densities in natural environments [4–8].

*Haemaphysalis longicornis* is a widely distributed tick species in China with a broad host range. The species originated in East Asia and has spread to New Zealand, Australia, and North America [9–11]. Furthermore, *H. longicornis* is a vector for numerous pathogens, including severe fever with thrombocytopenia syndrome virus, *Babesia* spp., *Theileria* spp., *Borrelia burgdorferi*, *Anaplasma phagocytophilum*, tick-borne encephalitis virus, and spotted fever group rickettsiae [12–16]. Nowadays, using chemicals, such as acaricides and repellents, to kill or deter ticks is a method of tick control. N, N-diethyl-meta-toluamide (DEET), sec-butyl 2-(2-hydroxyethyl) piperidine-1-carboxylate (icaridin), and N, N-diethyl-3-methylbenzamide (IR3535) are widely used chemical repellents that are effective against arthropods. In addition, some plant-derived compounds have demonstrated promising repellent effects against ticks [17–21]*.*

Ticks possess a highly developed olfactory sense that integrates chemosensory and neurological systems to enable rapid behavioral responses to external stimuli. The primary olfactory sense organ in ticks is Haller’s organ situated at the tarsal segments of the first pair of legs. This organ houses over 200 distinct types of olfactory receptor neurons that can detect various chemical cues, including sex pheromones, aggregation-attachment pheromones that signal the presence of other ticks, and signals from vertebrates. These sensory inputs are transmitted to the tick’s nervous system, thereby regulating its behavior [22, 23]. Despite advancements in our understanding of tick biology, the specific mechanisms through which ticks detect and process external odors remain poorly understood.

The transient receptor potential (TRP) channel is a nonselective cation channel whose activation is modulated by various external substances, including chemical compounds, temperature variation, and sounds. These stimuli can either directly open the TRP channel or initiate a cascade of biochemical responses [24–26]. TRP channels play crucial roles in regulating cell adhesion, polarity, proliferation, differentiation, and apoptosis [27–29]. In addition, TRP channels function as multimodal molecular sensors within cells, responding to factors such as temperature, tactile stimuli, osmolarity, pheromones, and taste [24, 30]. The TRP channels play a role in various repellent response mechanisms in arthropods [31, 32]; however, there have been no reports of TRP channels being found in ticks.

Given the increasing incidence of resistance to various repellents [33, 34], the present study aimed to enhance our understanding of the recognition mechanisms by which ticks respond to repellents. Our investigation focused on a range of tick repellents and their interactions with TRP channel proteins to identify new molecular targets. This research will contribute to improvements in public health and agricultural productivity.

## Methods

### Ticks and chemicals

A parthenogenetic strain of the tick *H. longicornis* was collected from Shanghai Wildlife Park, China, and established at the Shanghai Veterinary Research Institute, Chinese Academy of Agricultural Sciences, Shanghai, China. The ticks were kept in the laboratory at 25 °C and fed on New Zealand rabbits [35]. Ticks were collected during the nymphal stage at 10 days after molting. The following reagents were used in this study: cinnamaldehyde (Sinopharm Chemical Reagents, Shanghai, China), DEET (Macklin, Shanghai, China), Icaridin (Yuanye, Shanghai, China), and IR3535 (Yuanye, Shanghai, China).

### Avoidance response assay

In vitro assessment of tick repellency can be used for volatile compounds in the absence of host stimuli. Here, Y-tubes were used to observe the avoidance response of ticks [21, 36] (Y-tube is shown in Supplementary Fig. S1). In this study, 70 unfed nymphs were used for each test. Ethanol (95%) was used as the solvent for 20% DEET, 10% icaridin, 10% IR3535, and 2% cinnamaldehyde [18, 21, 37, 38]. Ticks exposed to each repellent comprised experimental groups, with 95% ethanol serving as the control treatment. A total of 10-µL droplets of each repellent were applied to a 1 cm filter paper and allowed to dry for 10 min (the avoidance test was performed when the ethanol was observed to evaporate on the filter paper) before being positioned at the top of a Y-tube that featured a nylon membrane. A total of six to eight independent trials were conducted. Statistical analysis was performed using Dunnett’s Multiple Comparison test, significant differences were indicated by *P* < 0.05, and data visualization was performed using GraphPad Prism 5 (GraphPad Software, CA, USA). The repellency rate was calculated using the following formula:

$${\text{Repellency}}{\mkern 1mu} \left( {{\% }} \right){\mkern 1mu} {\text{ = }}\frac{{{\text{Number}}{\mkern 1mu} {\text{of}}{\mkern 1mu} {\text{ticks}}{\mkern 1mu} \,{\text{in}}\,{\mkern 1mu} {\text{the}}\,{\text{control}}\,{\mkern 1mu} {\text{group - Number}}{\mkern 1mu} {\text{of}}{\mkern 1mu} {\text{ticks}}\,{\mkern 1mu} {\text{in}}{\mkern 1mu} \,{\text{the}}\,{\text{experimental}}{\mkern 1mu} \,{\text{group}}}}{{{\text{Number}}{\mkern 1mu} \,{\text{of}}{\mkern 1mu} \,{\text{ticks}}\,{\mkern 1mu} {\text{in}}{\mkern 1mu} \,{\text{the}}\,{\text{control}}{\mkern 1mu} \,{\text{group}}}}{{ \times 100\% }}$$

### Electroantennography

Electroantennography (EAG) is used to detect changes in the response of insects’ antennae to the external environment, revealing the olfactory mechanisms. The EAG assay is based on insect repellents [38–42]. The first pair of legs of the unfed nymphs were cut and quickly fixed onto the conductive adhesive of a metal fork and adjusted so that the Haller’s organs were exposed to the air (Supplementary Fig. S3). The odorants to be measured were diluted with paraffin oil, and 10 µl drops were placed on a 5 cm × 2.5 cm strip of filter paper and placed in a Pasteur pipette. The odorants were exposed to the air at a constant flow rate of 170 mL/min for 0.6 s, with an interval of 8 s between each administration. Recordings were made using an IDAC-2 and analyzed using EAGpro software. Each test was repeated six to eight times.

### cDNA constructs and sequence analysis of HL-TRP channel

Total RNA was extracted from 50 unfed nymphs using TRIzol reagent (Invitrogen, CA, USA) following the manufacturer’s protocol. Real-time polymerase chain reaction (RT-PCR) was performed with a cDNA synthesis kit (Vazyme, Nanjing, China) to generate a cDNA library. The genomic DNA was removed at 42 °C for 2 min and reverse transcribed at 37 °C for 15 min and at 85 °C for 5 s. The full-length cDNAs for HL-TRP channel were cloned using a PrimerSTAR Max DNA Polymerase kit (Takara, Osaka, Japan) from the tick cDNA library and subcloned into an pMD-18 T vector (Takara, Osaka, Japan). Oligonucleotide primers: F: 5′-ATGCAGTGCCGAAAGGATTC-3′ and R: 5′-CGCGAACGTTCGGCATAG-3′) were designed to amplify the open reading frame sequence of HL-TRP channel using Primer Premier 5. HL-TRP channel sequence analyses were performed using NCBI BLAST (BLAST, Basic Local Alignment Search Tool; nih.gov) and the SMART database (SMART; main page; embl.de)), and alignments were performed using Genetyx (version 6). Phylogenetic trees were built with MEGA5 using the neighbor-joining method.

### Subcellular localization

The HL-TRP subcloned into an EGFP-N1 vector (Addgene, MA, USA). Gene-specific primers: HL-TRP-EGFP-N1-F: 5′-GAACCGTCAGATCCGCTAGCATGCAGTGCCGAAAGGATTC-3′ containing restriction site (Nhe I:GCTAGC) and HL-TRP-EGFP-N1-R: 5′-ATTCGAAGCTTGAGCTCGAGTGCCGAACGTTCGCG-3′ containing restriction site (Xho I: CTCGAG). HEK 293 T cells were grown in DMEM (Gibco, NY, USA) containing 10% heat-inactivated fetal bovine serum (Gibco, NY, USA) and 2% penicillin–streptomycin (Beyotime Biotechnology, Shanghai, China) and were incubated at 37 °C with 5% CO_2_. Transfection was performed using lipo3000 (Invitrogen, CA, USA). The cells were placed on coverslips and allowed to grow for 24 h, then washed with phosphate-buffered saline (PBS), fixed with ethanol, permeabilized with 0.2% Triton X-100, washed with PBS, blocked with 1% (w/v) bovine serum albumin, and incubated with Na^+^, K^+^-ATPase α1 (1:50 dilution, CST, MA, USA), GFP tag monoclonal antibody (1:25 dilution, Proteintech, IL, USA), and primary antibody overnight at 4 °C. Subsequently, the cells were washed three times with TBST and incubated with Alexa 488-labeled goat anti-rabbit antibody (Invitrogen, CA, USA) and Alexa 594-labeled goat anti-mouse antibody (Invitrogen, CA, USA) for 1.5 h. Finally, they were washed once with TBST and incubated with DAPI (Hoechst 33342) (Invitrogen, CA, USA). The coverslips were scanned as multi-channel single planes using a ZEISS laser confocal microscope.

### Quantitative analysis of transcription

To analyze the effects of different repellents on tick HL-TRP channel, 95% ethanol containing 10% icaridin, 20% DEET, 2% cinnamaldehyde, or 10% IR3535 was applied on a 1-cm diameter piece of filter paper, and approximately 50 unfed nymphs were stimulated for 30 min and then snap-frozen in liquid nitrogen to extract the RNA.

For the analysis of expression patterns of the HL-TRP channel, 500 ticks at different developmental stages (egg, unfed larvae, engorged larvae, unfed nymphs, engorged nymphs, unfed adults, fed adults (adult ticks fed for 3–4 days), and engorged adults) were collected. The 200 unfed adults were dissected to obtain tissues from the legs, synganglion, midgut, ovaries, salivary glands, and fat bodies.

The samples were treated with TRIzol reagent to obtain RNA and reverse transcribed into cDNA using a HiScript® III RT SuperMix for qPCR (+ gDNA wiper) kit (Vazyme, Nanjing, China).

The cycling schedule was 95 °C for 30 s, followed by 40 cycles of 95 °C for 5 s and 60 °C for 30 s. Gene expression was examined using an ABI QuantStudio Q5 quantitative PCR instrument (Thermo Fisher Scientific, MA, USA). Elongation factor-1 (ELF1A) was used as the internal reference [38, 43], and the primers used for qRT-PCR were as follows: HL-TRP-qPCR-F: 5′-GATCCTGCTCACGGTTCTGT-3′; HL-TRP-qPCR-R: 5′-GGTGATGGCGTTAAGAGGGG-3′; ELFIA-F: 5′-CGTCTACAAGATTGGTGGCATT-3′; and ELFIA-R: 5′-CTCAGTGGTCAGGTTGGCAG-3′. Data was analyzed using the 2^−ΔΔCt^ method. RNA interference statistical analysis was conducted using Student’s *t*-test, and significant differences were indicated by *P* < 0.05, *P* < 0.01, and *P* < 0.001. Expression patterns statistical analysis was conducted using Dunnett’s Multiple Comparison test, and significant differences were indicated by *P* < 0.05. Each sample had three independent biological replicates and three technical replicates.

### Identification of HL-TRP channel by in situ hybridization

The probes were designed specifically on the basis of the HL-TRP sequence: TGCGGGAGTTACACGAATAGGGAAT; ACGCAAGTCGTAGACACTCGATGATTC; TTATTTCAGGCTTCAAGACTGTTTCGA; ACTGGAGTGTCCGTATGCATGAAAAT; and TGATGGGAGCAGTCCACATTAGGTT. Paraffin sections were prepared after the first pair of legs was removed and fixed. The sections were digested with proteinase K (20 µg/ml) and washed three times with PBS at 37 °C. Pre-hybridization was performed at 37 °C for 1 h, followed by the addition of 500 nM probes and overnight hybridization at 42 °C, after which the sections were washed three times with saline sodium citrate buffer (SSC). The samples were maintained in buffer at 40 °C for 45 min and then washed three times with SSC. Drops of signal hybridization solution containing anti-DIG antibody were applied, and the samples were incubated in a humid chamber at 40 °C for 45 min before washing three times with SSC. DAPI staining was conducted under light-avoidance conditions, and the slices were blocked with an anti-fluorescence quenching agent. Images were captured for analysis using orthogonal fluorescence microscopy. The DAPI-stained nuclei appeared blue under ultraviolet illumination, while structures stained with FAM (488) appeared green. The reagents were obtained from Servicebio (Wuhan, China).

### RNAi-mediated HL-TRP channel gene knockdown

Primers were designed to synthesize double-stranded RNA (dsRNA) using the HL-TRP channel sequence as a template (Table 1). The dsRNAs were synthesized using a T7 RiboMAX Express RNAi System kit (Promega, WI, USA) following the manufacturer’s instructions. PCR amplification was conducted using specific primers that incorporated the T7 promoter sequence. The amplification was performed using a PCR program of 94 °C for 3 min, followed by 30 cycles consisting of 94 °C for 30 s, 57 °C for 30 s, and 72 °C for 1 min. After 5 min at 72 °C, the samples were stored at 4 °C. After that, the dsRNA was purified and mixed in RNase-free ddH_2_O to check its purity and concentration. The samples were kept in a freezer at 80 °C.

**Table 1:** Primer sequences used for RNAi

Gene name	Primer sequence
RNAi-Lusiferase-F1	5′-GGATCCTAATACGACTCACTATAGGGCTTCCATCTTCCAGGGATACG-3′
RNAi-Lusiferase-R1	5′-CGTCCACAAACACAACTCCTCC-3′
RNAi-Lusiferase-F2	5′- GCTTCCATCTTCCAGGGATACG-3′
RNAi-Lusiferase-R2	5′-GGATCCTAATACGACTCACTATAGGCGTCCACAAACACAACTCCTCC-3′
RNAi-HL-TRP-F1	5′-TAATACGACTCACTATATGCAGTGCCGAAAGGATTC-3′
RNAi-HL-TRP-R1	5′-TTCCTTGCAGGACGAGCAC-3′
RNAi-HL-TRP-F2	5′-ATGCAGTGCCGAAAGGATTC-3′
RNAi-HL-TRP-R2	5′-TAATACGACTCACTATAGGTTCCTTGCAGGACGAGCAC-3′

The dsRNAs were quantified and diluted with water to 1.5 μg/μl. Equal amounts of Luciferase were used as a control [44, 45]. The surfaces of the unfed nymphs were cleaned and mounted on double-sided tape with the dorsal side up. Each nymph was injected with 9.2 nL dsRNA using the “Nanoject II” system (Drummond Scientific, PA, USA). Each group was injected with 60 unfed nymphs in three biological replicates. The injected ticks were kept in clean tubes and placed at room temperature for 48 h. After 48 h, nymphs that could move autonomously were used in electroantennography and avoidance response assay.

### Prediction of the binding site of HL-TRP channel to repellents

AlphaFold3 was used to predict the crystal structures of HL-TRP channel proteins, and these were then processed using the Protein Preparation Wizard module of Schrödinger software (Schrödinger, NY, USA). The four repellents were modeled in two dimensions (2D) and three dimensions (3D) using Schrödinger’s LigPrep module. The SiteMap module was used to predict the ideal binding site, and the Receptor Grid Generation module was used to choose the best enclosing box. Following molecular docking, the bound active sites were evaluated using MM-GBSA calculations and the MM-GBSA dG bind computation of the free energy.

## Results

### Repellents are effective against *H. longicornis* at the nymph stage

**Fig. 1:**
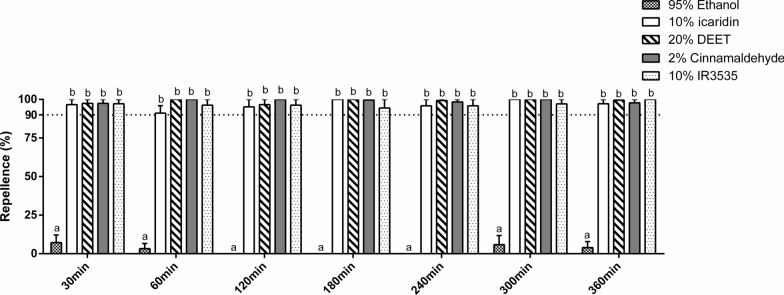
Repellency of various treatments within 6 h in nymphal tick (**** *P* < 0.0001)

The repellent efficacy of the four treatments against nymphs was recorded using 95% ethanol as a control. The average repellency of 95% ethanol at various time intervals (30 min; 60 min; 120 min; 180 min; 240 min; 300 min; and 360 min) was recorded as 7.2%; 3.3%; 0%; 0%; 0%; 0%; and 0%, respectively. In contrast, the average repellency of 10% icaridin at the same time intervals was 96.7%; 91.1%; 95.2%; 100%; 95.8%; 100%; and 97.2%. The average repellency of 20% DEET was similarly higher than 90%; with values of 97.4%; 100%; 96.7%; 100%; 99.3%; 100%; and 99.5% across the specified periods. In addition, the average repellency of 2% cinnamaldehyde was measured as 97.4%; 100%; 100%; 99.6%; 98.3%; 100%; and 97.8%. Lastly, the average evasion rates of 10% IR3535 at the corresponding time intervals were 97.2%; 96.3%; 96.3%; 94.4%; 95.8%; 97.1%; and 100% (Fig. 1).

### Identification of the HL-TRP channel in *H. longicornis*

**Fig. 2:**
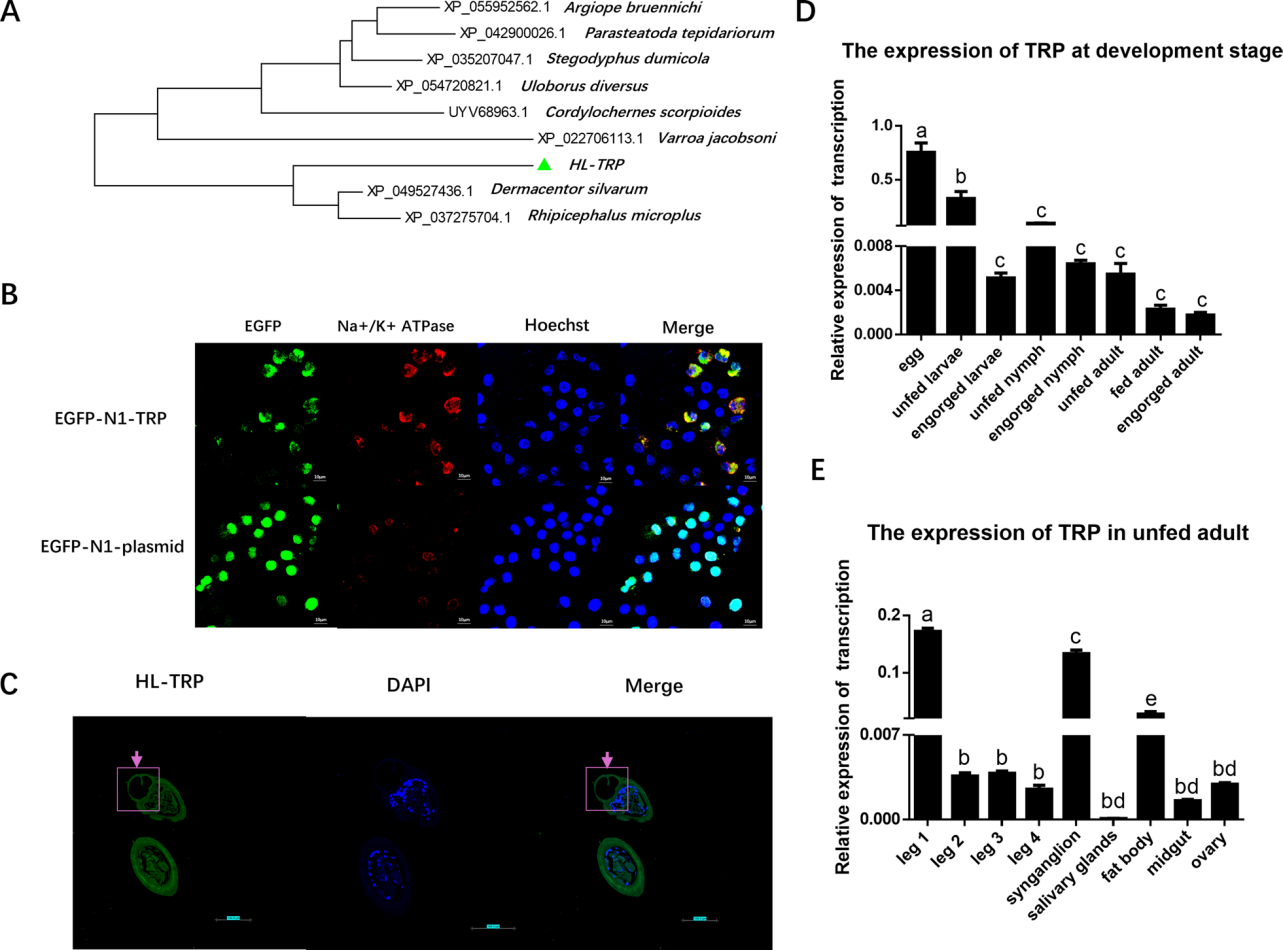
Identification of the HL-TRP channel. **A** The relationship of HL-TRP channel with other species from a maximum likelihood phylogeny; HL-TRP is marked with red triangles. **B** Immunofluorescence of HL-TRP in HEK293T; green fluorescence: HL-TRP; red fluorescence: Na^+^, K^+^-ATPase α1, cell membrane; blue fluorescence: cell nuclei; scale bar = 10 μm. **C** The expression of HL-TRP in the first pair of tick legs by in situ hybridization. Pink arrow and box mark Haller’s organ; green fluorescence: HL-TRP; blue fluorescence: cell nuclei; scale bar = 50 μm. **D** mRNA expression of the HL-TRP channel at different developmental stages. Different letters indicate significant differences (*P* < 0.05). **E** Transcription analysis of the HL-TRP channel in different tissues during an unfed stage. Different letters indicate significant differences *(P* < 0.05)

Putative TRP channel genes were screened in the *H. longicornis* complementary DNA (cDNA) library using the RACE technique. The HL-TRP channel (no. PQ189785.) encodes a protein consisting of 906 amino acids and features seven putative transmembrane domains, along with the LSDAT_euk and trp structural domains, categorizing it within the TRP molecular family (Supplementary Fig. S2). Notably, the HL-TRP channel exhibits greater similarity to those of other tick species than to those of other arthropods (Fig. 2A). To further investigate HL-TRP properties, its heterologous expression was performed. HL-TRP channel is co-localized with the cell membrane as well as present in the cytoplasm (Fig. 2B). In addition, in situ hybridization on a cross-section of first pair of tick legs revealed widespread signals from HL-TRP channel located on the Haller’s organ (Fig. 2C). Its presence throughout the tick’s life cycle and organization was confirmed through RT-qPCR, demonstrating its expression in *H. longicornis*. Transcript levels were higher during the initial phases of development, particularly in the eggs, as well as in the first set of legs and synganglion of unfed adults. (Fig. 2D, E).

### The HL-TRP channel is required for the recognition of repellents

**Fig. 3:**
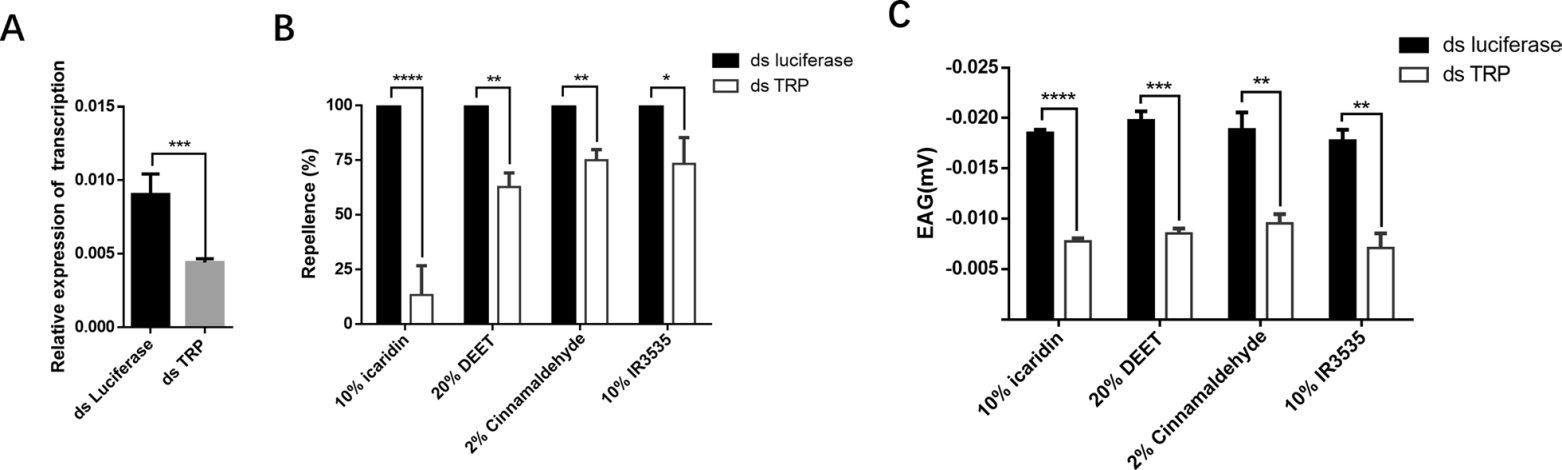
The HL-TRP channel acts as a repellent receptor. **A** Transcription levels of RT-qPCR confirmation of the HL-TRP channel RNAi. **B** Tests of different repellents after HL-TRP channel interference. **C** EAG tests for different repellents after HL-TRP channel interference. * *P* < 0.05, ** *P* < 0.01, *** *P* < 0.001, and **** *P* < 0.0001

We investigated the relationships between various sources of chemical and plant-origin repellents and HL-TRP channel. RNAi (Fig. 3A) of nymphs revealed that the control group’s repellency remained at 100% across all tested concentrations: 10% icaridin, 20% DEET, 2% cinnamaldehyde, and 10% IR3535. In contrast, the experimental group’s percentages decreased to 6.7%, 62.7%, 75%, and 73.3% for the same repellents at the corresponding time points (Fig. 3B). Furthermore, there was a near-synchronization between the repellent effect and the EAG response following RNAi. The EAG results for the experimental group were significantly lower than those of the control group for 10% icaridin, 20% DEET, 2% cinnamaldehyde, and 10% IR3535, yielding values of −0.0186, −0.0198, −0.0189, and −0.0178 mV, respectively. In comparison, the EAG values for the control group were −0.0078, −0.0086, −0.0096, and −0.0071 mV for the same repellents (Fig. 3C).

### The TYR783 site is a key binding site for repellents

**Fig. 4:**
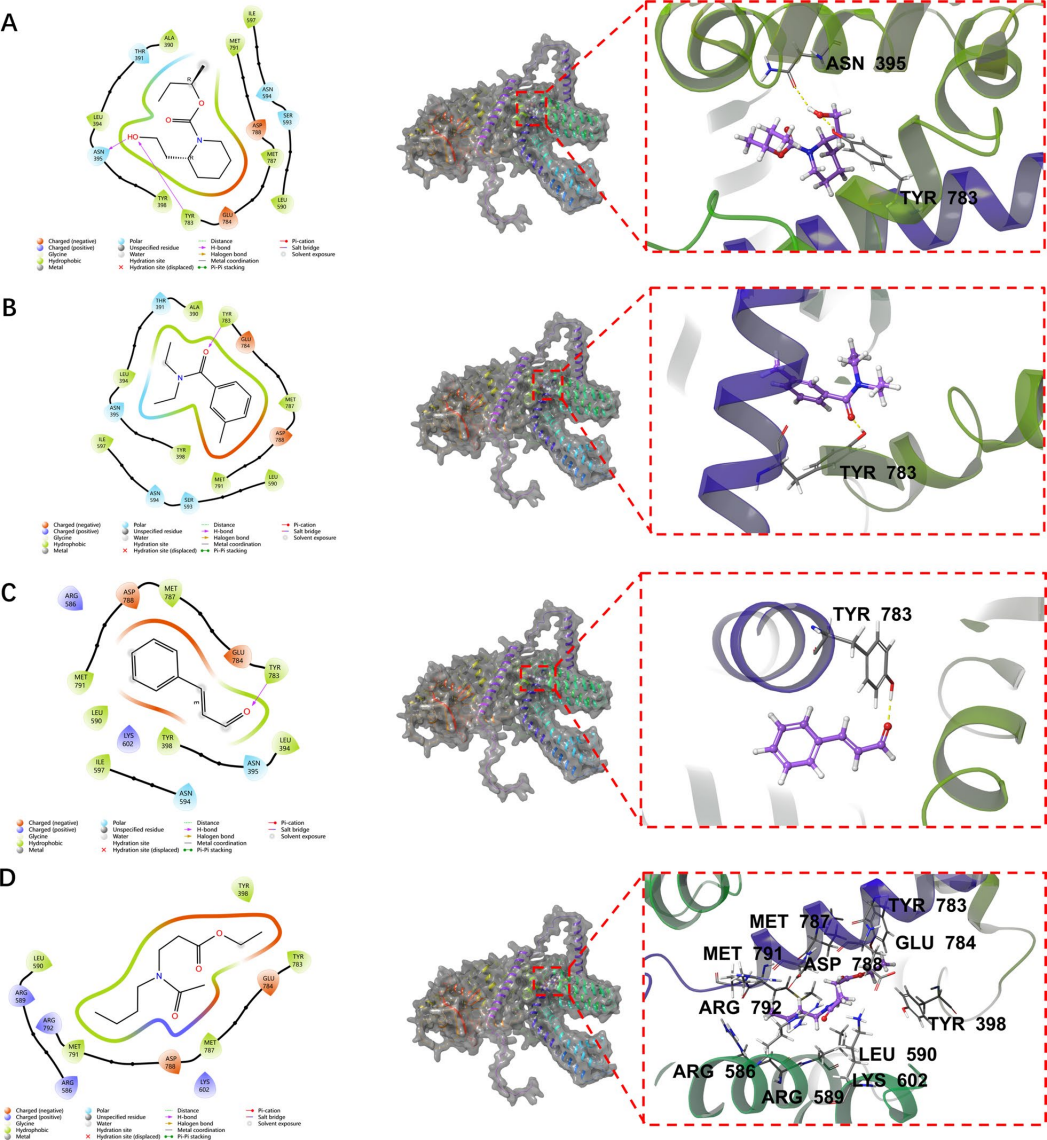
Predictions of the binding sites of different repellents to HL-TRP channel. **A** Icaridin, **B** DEET, **C** cinnamaldehyde, and **D** IR3535. The left side shows a 2D bonded model and the right side a 3D bonded model

We used molecular docking prediction and protein modeling to further clarify the relationship between the HL-TRP channel and different repellents. An XP GScore value of less than −6 suggests that the ligand exhibits stable protein binding properties. In addition, an MM-GBSA dG bind value of less than −30 kcal/mol indicates weak binding free energy, further supporting the notion that the ligand maintains stability in its interaction with the protein. The predicted binding capacity of HL-TRP and repellents is presented in Table 2. Docking studies revealed that icaridin penetrates deeply into the active pocket of the HL-TRP channel protein, where the protein residues LEU394, TYR398, TYR783, and MET791 have hydrophobic interactions with icaridin. In addition, the ligand forms hydrogen bonds with the protein residues ASN395 and TYR783 (Fig. 4A). Similar to icaridin, both DEET and cinnamaldehyde penetrated deeply into the active pocket of the HL-TRP channel protein. The protein residues ALA390, TYR783, and MET787 contribute to hydrophobic interactions with DEET, while TYR783 and LEU394 provide hydrophobic interactions with cinnamaldehyde. In addition, both DEET and cinnamaldehyde could form hydrogen bonds with the protein residue TYR783 (Fig. 4B, C). Nevertheless, IR3535 appeared capable of entering the active pocket of the HL-TRP channel protein. Although the protein residues LEU590 and MET791 exerted a hydrophobic pull on cinnamaldehyde, there was no non-covalent interaction (Fig. 4D).

**Table 2:** XP&MM-GBSA analysis of HL-TRP and repellent

Compound	XP Gscore	MM-GBSA dG Bind(kcal/mol)	Binding interaction
Icaridin	−5.361	−29.90	Stable
DEET	−4.882	−36.87	More stable
Cinnamaldehyde	−3.994	−32.30	More stable
IR3535	−2.151	−26.98	Unstable

## Discussion

TRP channels are prevalent in arthropods, and they play crucial roles in various sensory processes. TRP channels are classified into seven distinct classes on the basis of their amino acid sequences: TRPA (Ankyrin), TRPC (Canonical), TRPM (Melastatin), TRPML (Mucolipin), TRPN (Normpc), TRPP (Polycystic), and TRPV (Vanilloid). Except for TRPN, all classes have been conserved throughout evolution. TRPs have been identified across nearly all species within the insect class of Arthropoda, with each subfamily serving unique functions [46]. TRPA is linked to temperature and chemosensation, sensory modalities that influence circadian rhythms and developmental processes, and it is also responsive to humidity and light [47]. TRPC, TRPN, and TRPV are involved in modulating a diverse array of mechanosensory sensations, including motion detection, hearing, and humidity perception, and they also play a role in reproductive behaviors such as egg-laying [48–51]. In addition, TRPM is associated with metabolic processes, while impairment of TRPML has been linked to locomotor disorders. TRPP has significant effects on reproductive functions [52, 53].

Arthropods’ responses to repellents have been linked to the TRP channel family. In beetles, TRPM mediates the repellent activity of mint derivatives [54]; carvacrol inhibits TRPM7; and TRPA1 and TRPM are necessary for menthol-induced repellent behavior in *Drosophila* [55, 56]. *Varroa destructor* TRPA1 is strongly activated by eight representative plant-derived compounds, namely 1,8-eucalyptol, β-citronellol, 2-undecanone, myrtle aldehyde, nerolidol, methyl jasmonate, carvacrol, and α-pinitol [32, 57]. Similarly, TRP1 is potentiated by citronella derivatives in *Mesobuthus martensii*[58]. The primary constituents of Guyana essential oils, β-laurene and 2-undecanone, may interact with lipid binding in *Myzus persicae* and activate/regulate TRP channels [59]. RNAi studies have demonstrated that the HL-TRP channel plays a significant role in the repellent response and the recognition of repellents such as DEET, icaridin, and cinnamaldehyde. Predictive analyses have further indicated that the differences in their effectiveness closely followed the predictions from the binding assays. Notably, the predicted binding sites were remarkably similar across tick species (Supplementary Fig. S4). These findings suggest that the HL-TRP channel functions in ticks as a broad-spectrum repellent receptor. Indeed, we attempted to utilize exogenous expression and patch clamp technology to monitor the potential changes in the HL-TRP channel induced by four repellents. However, we were unable to obtain satisfactory experimental results owing to operational instability.

Here, the HL-TRP channel we identified may have a dual role, as it contained both TRPM and TRPV structural domains. TRPM channels have been implicated in reproductive processes in arthropods, where Ca^2+^ influx facilitates mechanical stimulation that enhances laying eggs [60, 61]. In addition, TRPM channels play a crucial role in regulating Zn^2+^ homeostasis during larval development, mediating the molecular mechanisms through which phosphoinositide 3-kinase (IPI3K) influences cellular growth [62]. Furthermore, TRPM channels activate class III dendritic sensory neurons, thereby inducing whole-body contractions and modulating cold sensation in *Drosophila* larvae [63]. TRPM proteins are also involved in the homeostasis of Mg^2+^ and Zn^2+^, and they are expressed in the Malpighian tubules where they function to scavenge electrolytes and toxic substances from the hemolymph [62, 64]. TRPV channels in arthropods represent critical targets for insecticides. In invertebrates, these channels can be categorized into four types: Nanchung, inactive, Ocr-2, and Osm-9. In *Drosophila*, both Nanchung and inactive types are present. When exposed to insecticides, the Nanchung and inactive forms cooperate to form ion channels that facilitate the selective transport of calcium ions, thereby generating cationic currents in neurons. This process effectively translates external physical signals into biochemical signals in vivo. Furthermore, insecticides disrupt the feeding capabilities of insects through their interaction with TRPV channels [65]. TRP channels have been identified as the targets of various insecticides, including imidacloprid, pirfluoroquinoline, and nicotinamide [66, 67].

Among the four repellents studied, our prediction was that IR3535 does not interact with HL-TRP channel. In fact, we noticed that transcript levels were reduced and there was a drop in how effective the repellent was, but it was not entirely eliminated after RNA interference (RNAi). It might be necessary to enhance the efficiency of RNAi in the future to solve this problem. Furthermore, this might indicate that the HL-TRP channel is not the only receptor for the other three repellents. TRPV channel Nanchung and the TRPA channel Water Witch can form a complex that activates the response to reactive electrophiles such as allyl isothiocyanate and cinnamaldehyde [68]. In *Drosophila*, a dual pathway is necessary for the response to citronellal; specifically, OR83b is essential for generating citronellal-induced action potentials, while the Gq/PLC/TRPA1 pathway appears to modulate the frequency of these action potentials through the activation of the BK channel [31]. The exploration of synergistic molecules in ticks remains an area that requires further investigation.

## Conclusions

This study discovered that the tick TRP channel is essential for identification of the repellents icaridin, DEET, and cinnamaldehyde, and we also predicted the same location, TYR783, where the various repellents are identified. This not only deepens our understanding of the repellent recognition mechanisms in ticks but also provides new ideas for future integrated control strategies.

## Supplementary Information

Additional File 1: Fig. S1. Y-tube device diagram.

Additional File 2: Fig. S2. Predicted amino acid sequence encoded by the HL-TRP channel. The amino acid sequence of the full-length HL-TRP. Putative transmembrane domains (TMs) are shown in black; LSDAT_euk is underlined in green; and TRP is underlined in red. LSDAT_euk is the SLOG for TRPM, and trp also contains the TRPV structure field.

Additional File 3: Fig. S3. Operational diagram of Electroantennography. The red circle marks the location of Haller's organ.

Additional File 4: Fig. S4. Sequence comparison of the HL-TRP channel and TRP sequences of other ticks at amino acids 748–805 (containing TYR783). Identical residues in the sequences are indicated by black boxes.

## Data Availability

No datasets were generated or analyzed during the current study.
