# Supplementary figures and images for: HL-TRP channel is required for various repellents for the parthenogenetic Haemaphysalis longicornis
Source: Parasit Vectors. 2025 Apr 14;18:139. doi: 10.1186/s13071-025-06776-1 (PMC11995592; doi:10.1186/s13071-025-06776-1)

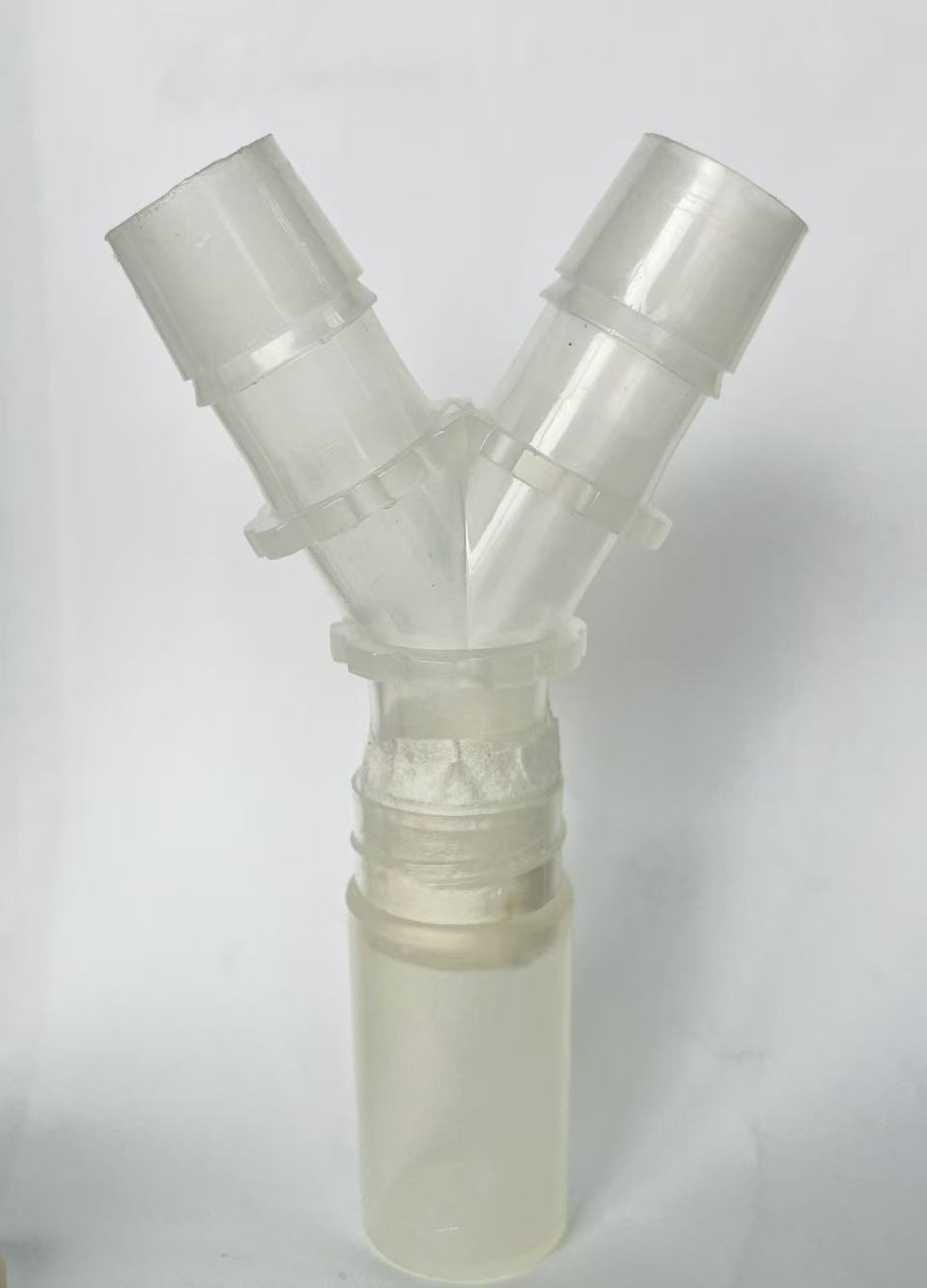

Supplement: Supplementary file 1 — Additional File 1: Fig. S1. Y-tube device diagram. [file 13071_2025_6776_MOESM1_ESM.tif]

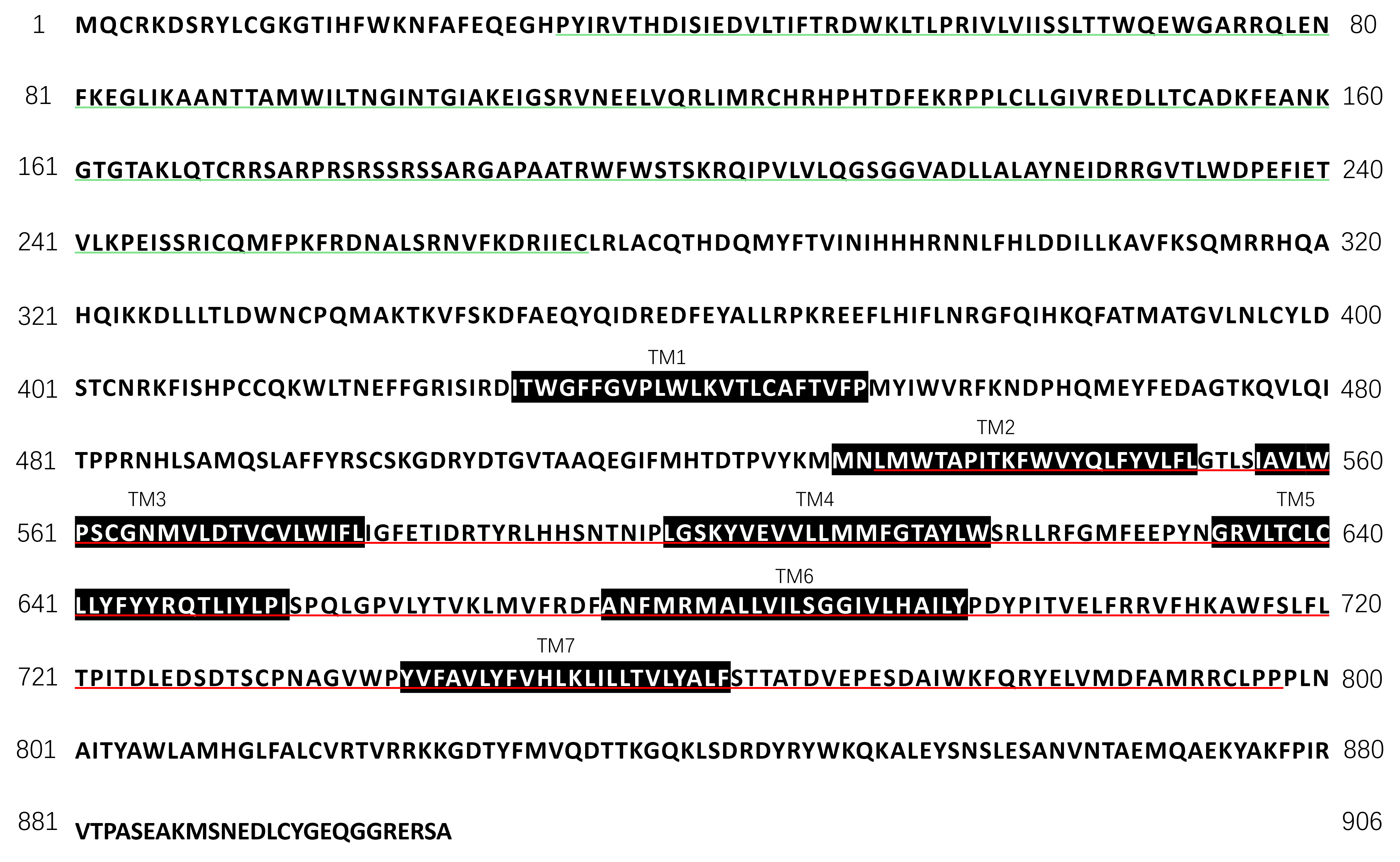

Supplement: Supplementary file 2 — Additional File 2: Fig. S2. Predicted amino acid sequence encoded by the HL-TRP channel. The amino acid sequence of the full-length HL-TRP. Putative transmembrane domains (TMs) are shown in black; LSDAT_euk is underlined in green; and TRP is underlined in red. LSDAT_euk is the SLOG for TRPM, and trp also contains the TRPV structure field. [file 13071_2025_6776_MOESM2_ESM.tif]

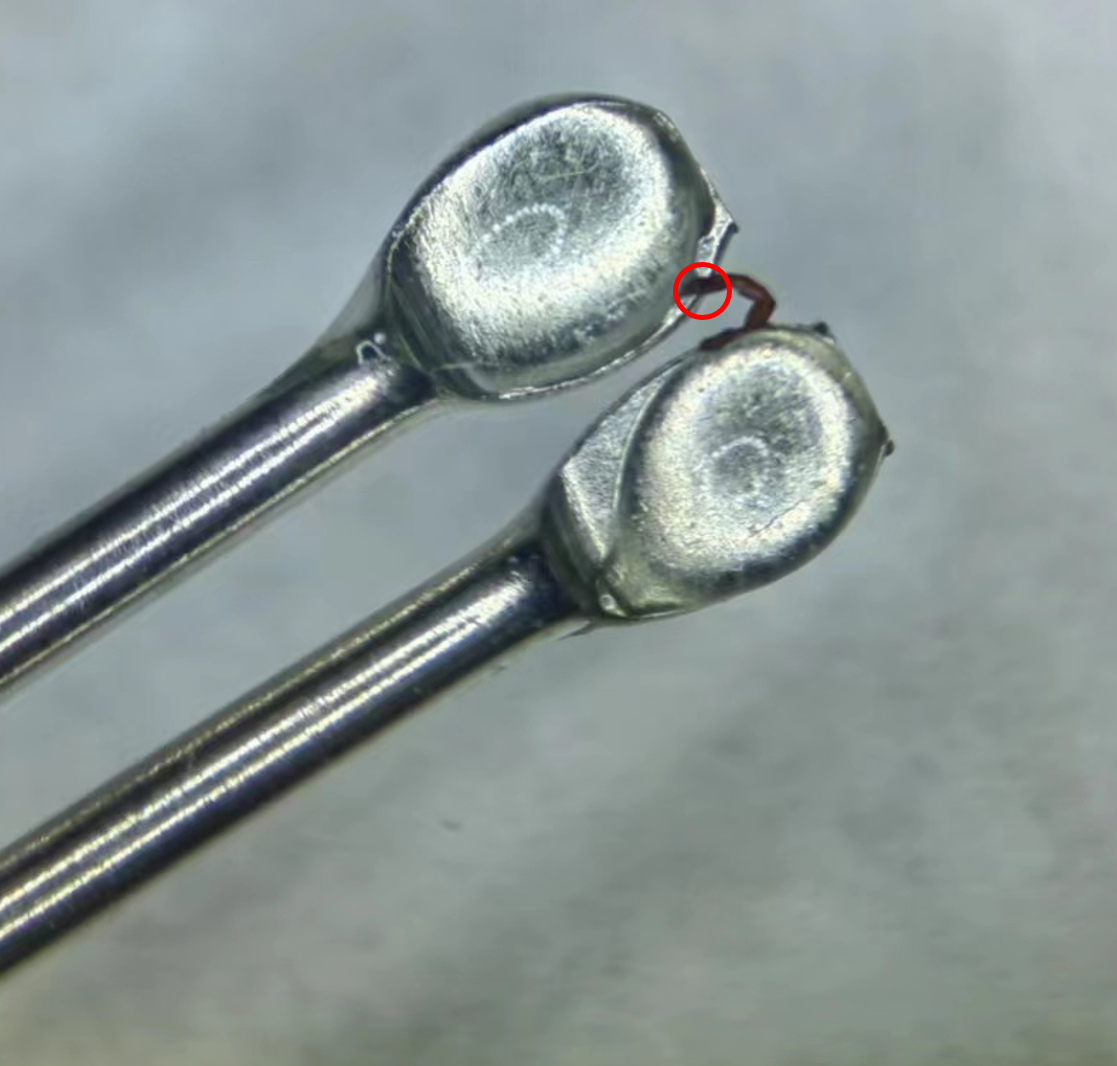

Supplement: Supplementary file 3 — Additional File 3: Fig. S3. Operational diagram of Electroantennography. The red circle marks the location of Haller's organ. [file 13071_2025_6776_MOESM3_ESM.tif]

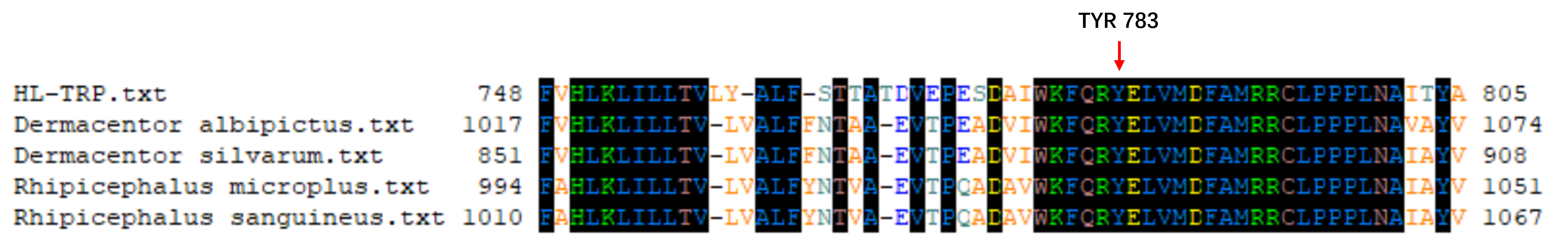

Supplement: Supplementary file 4 — Additional File 4: Fig. S4. Sequence comparison of the HL-TRP channel and TRP sequences of other ticks at amino acids 748–805 (containing TYR783). Identical residues in the sequences are indicated by black boxes. [file 13071_2025_6776_MOESM4_ESM.tif]
